# Supplementary material for: Air pollution impacts from warehousing in the United States uncovered with satellite data
Source: Nat Commun. 2024 Jul 24;15:6006. doi: 10.1038/s41467-024-50000-0 (PMC11269699; doi:10.1038/s41467-024-50000-0)
Supplement: Supplementary file 1 — Supplementary Information [file 41467_2024_50000_MOESM1_ESM.pdf]

Supplementary Information for

Air pollution impacts from warehousing in the United States uncovered with satellite data

Gaige Hunter Kerr[1], Michelle Meyer[2], Daniel L. Goldberg[1], Joshua Miller[2], & Susan C. Anenberg[1]

[1] Department of Environmental and Occupational Health, George Washington University, Washington, DC, USA 20052
[2] International Council on Clean Transportation, Washington, DC, USA 20005

Contents of Supplementary Information:
Supplementary Figures S1 to S14

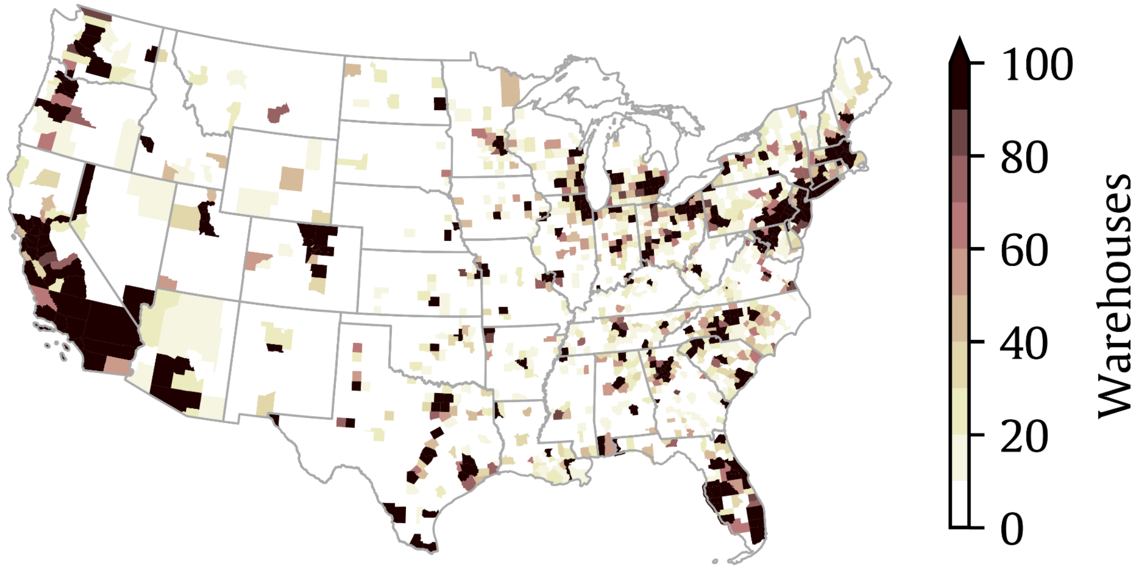

Figure S1. The total number of warehouses in U.S. counties as of 2021.

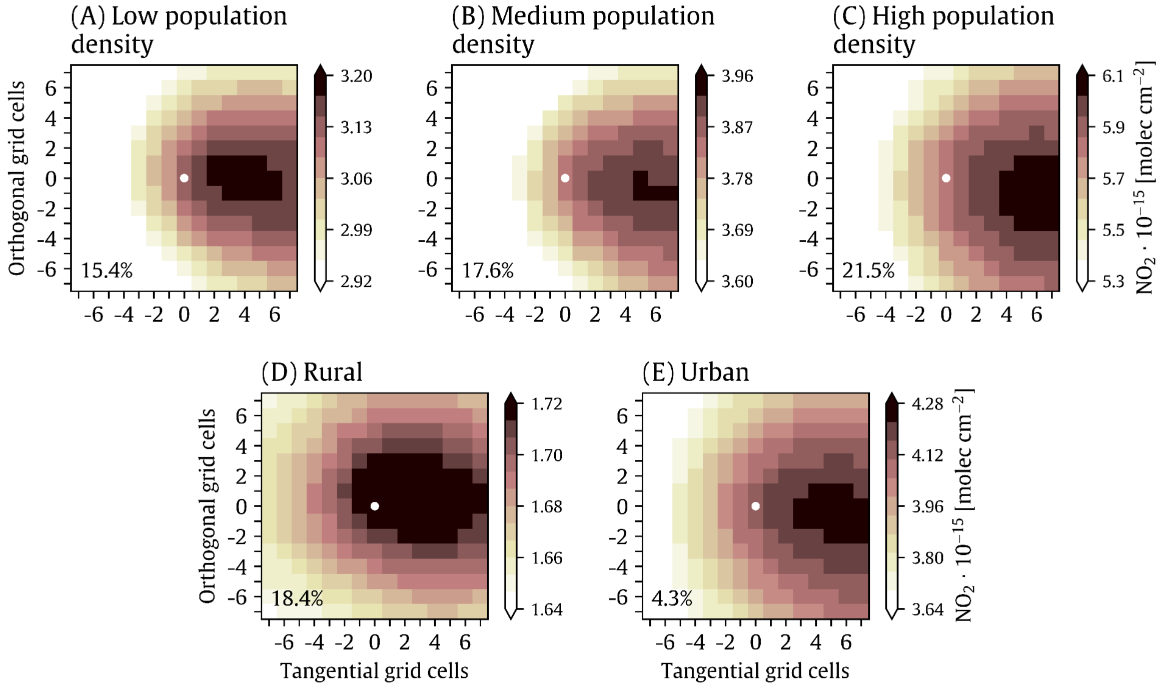

Figure S2. **Near-warehouse NO_2 enhancements for different population density and urban-rural environments.** Annual average 2021 TROPOMI NO_2 composited over warehouses located in census tracts in **(A)** low, **(B)** medium, and **(C)** high population density environments. The white scatterpoint corresponds to the location of the warehouses. In **(D)**, composites were formed with warehouses located in counties that were not within Metropolitan Statistical Areas (MSAs), as defined by the U.S. Census Bureau. **(E)** represents a composite of warehouses located within counties containing MSAs. Inset text in the lower left corner represents the percent NO_2 change between levels at the windward orthogonal edge of the composite and the maximum levels in the composite.

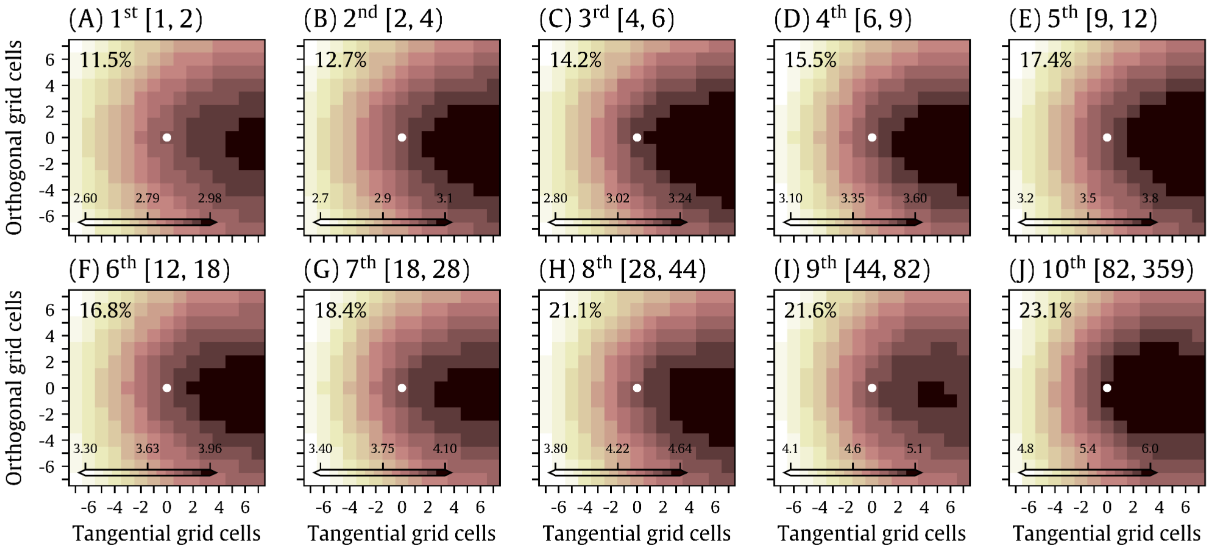

Figure S3. **Near-warehouse NO_2 enhancements for different levels of warehouse clustering.** Annual average 2021 TROPOMI NO_2 composited over warehouses by deciles of warehouse clustering. The decile is denoted in the subplots' titles along with the range of warehouses comprising the clustering using interval notation (e.g., [1,2) represents composited NO_2 when only a single warehouse is located in a census tract; [2,4) represents a composite when 2 or 3 warehouses are in a census tract, etc.). The white scatterpoint corresponds to the location of the warehouses. We note that the values shown in the composites and defined in the inset colorbars are in units of molecules cm^{-2} and have been scaled by 10^{-15}. Inset text in the upper left corner represents the near-warehouse NO_2 enhancement–that is, the percent NO_2 change between levels at the windward orthogonal edge of the composite and the maximum levels in the composite. Specifically, **(A)** refers to a composite formed for 1-2 warehouses per tract; **(B)** for 2-4; **(C)** for 4-6; **(D)** for 6-9; **(E)** for 9-12; **(F)** for 12-18; **(G)** for 18-28; **(H)** for 28-44; **(I)** for 44-28; and **(J)** for 82-359.

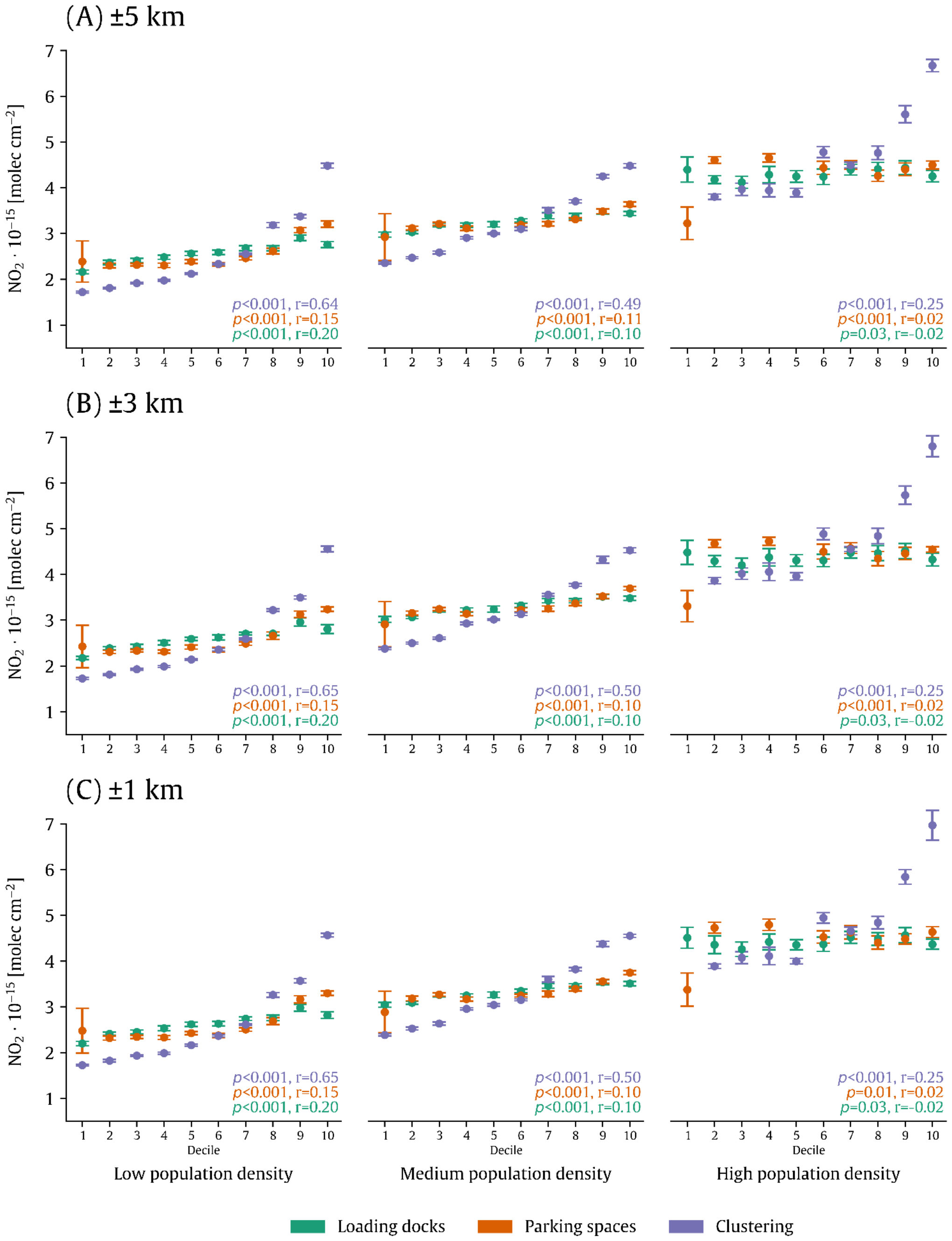

Figure S4. The role of warehouse characteristics in near-warehouse NO_2 enhancements for different near-warehouse definitions. Median NO_2 levels (scatterpoints) and the 95% confidence interval (vertical bars) formed by discretizing warehouses that comprise the composite into deciles based on their number of loading docks, parking spaces, and clustering for different population density classifications. **(A)** Represents NO_2 levels when the definition of "near warehouse" is restricted from the ±7 grid cells value used in the main text to ±5 grid cells, **(B)** to ±3 grid cells, and **(C)** to ±1 grid cells. Inset text indicates the *p*-value (*p*) and correlation coefficient (*r*) of the relationship between NO_2 and property characteristics but calculated using the full dataset, rather than the deciles. Median NO_2 levels and the associated confidence interval for the top clustering decile in high population density areas are out of frame and are shown alongside the plot.

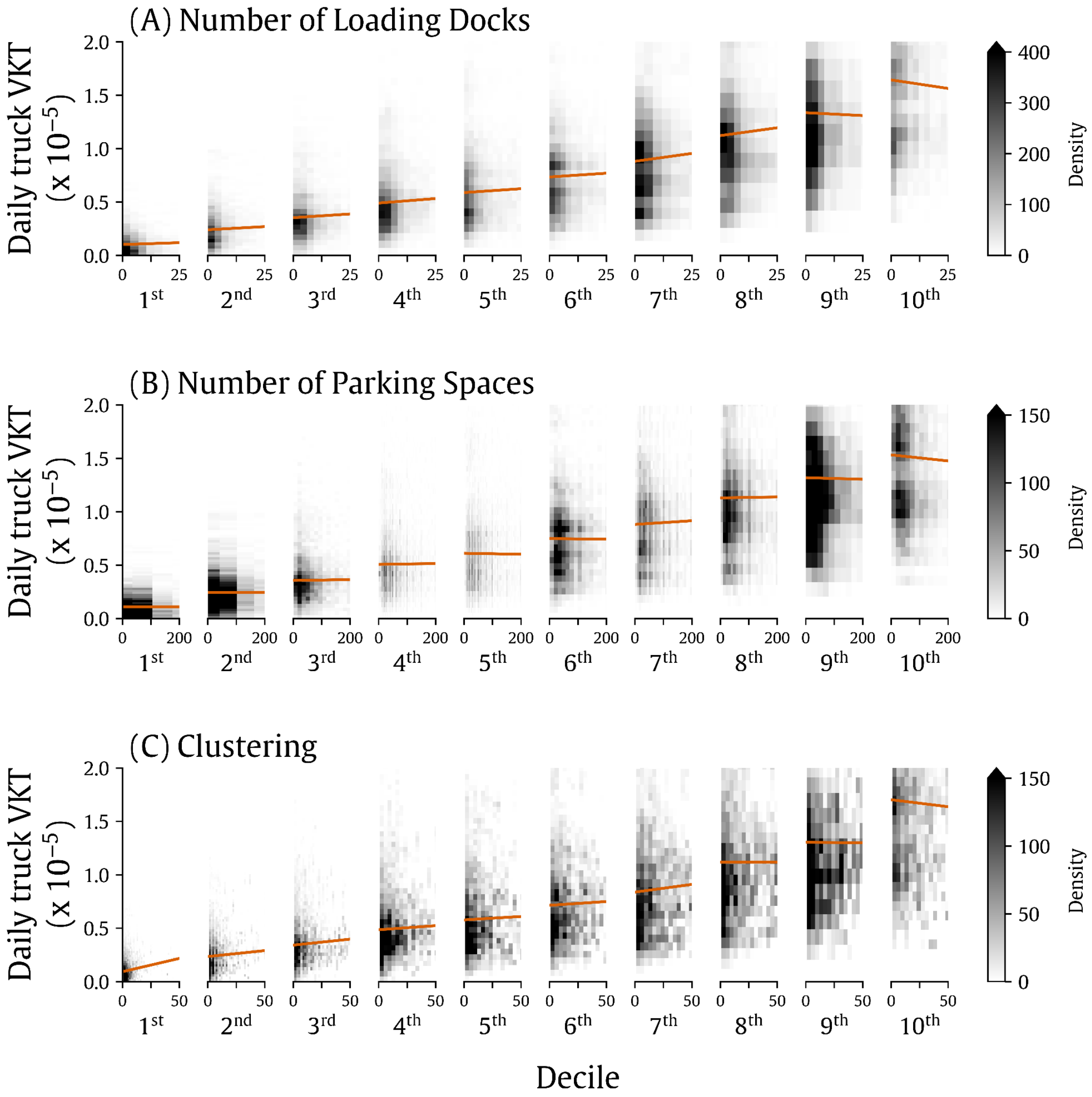

Figure S5. Relationships between daily truck vehicle kilometers traveled (VKT) and warehouse characteristics. Scatterplots colored by density showing near-warehouse daily truck VKT versus **(A)** the number of loading docks and **(B)** parking spaces at warehouses and **(C)** the number of warehouses in each census tract. Each of the ten panels in **(A)-(C)** represent different decile bins of daily total VKT, indicated by the cardinal numbers below the subplots. The number of loading docks and parking spaces or number of clustered warehouses are also indicated above the decile label. Orange lines represent linear regressions, and the slopes of these lines are visualized in Figure 3A in the main text.

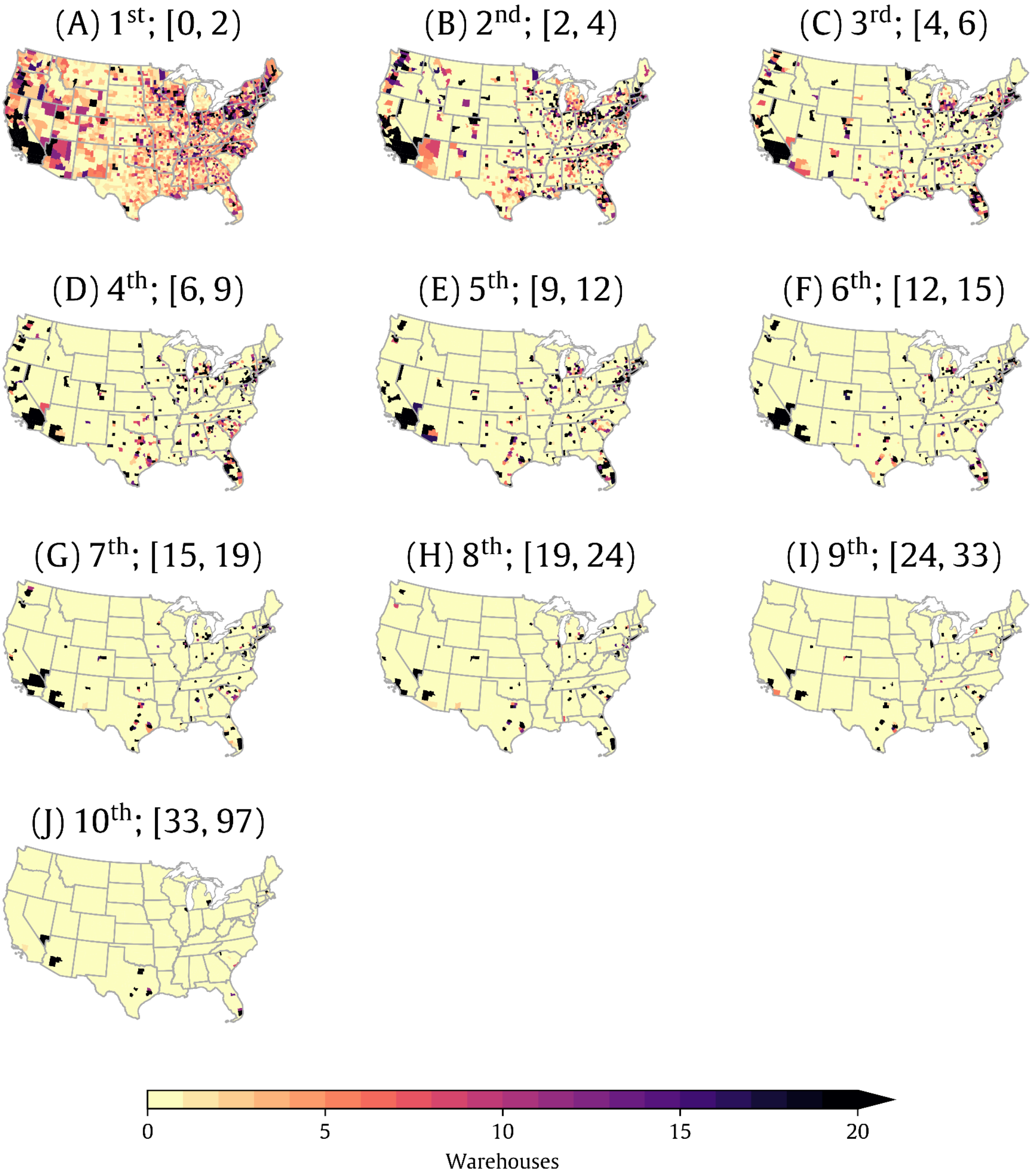

Figure S6. County warehouse counts discretized by underlying daily total VKT. The number of warehouses in each U.S. county based on their nearby daily total VKT, binned into deciles. The deciles are indicated by the cardinal numbers in subplots' titles along with the daily total VKT ranges that comprise the decile (in millions of kilometers) using interval notation. Specifically, **(A)** corresponds to 0 to 2,000,000 kilometers; **(B)** to 2,000,000 to 4,000,000 kilometers; **(C)** to 4,000,000 to 6,000,000; **(D)** to 6,000,000 to 9,000,000; **(E)** to 9,000,000 to 12,000,000; **(F)** 12,000,000 to 15,000,000; **(G)** 15,000,000 to 19,000,000; **(H)** 19,000,000 to 24,000,000; **(I)** to 24,000,000 to 33,000,000; and **(J)** to 33,000,000 to 97,000,000.

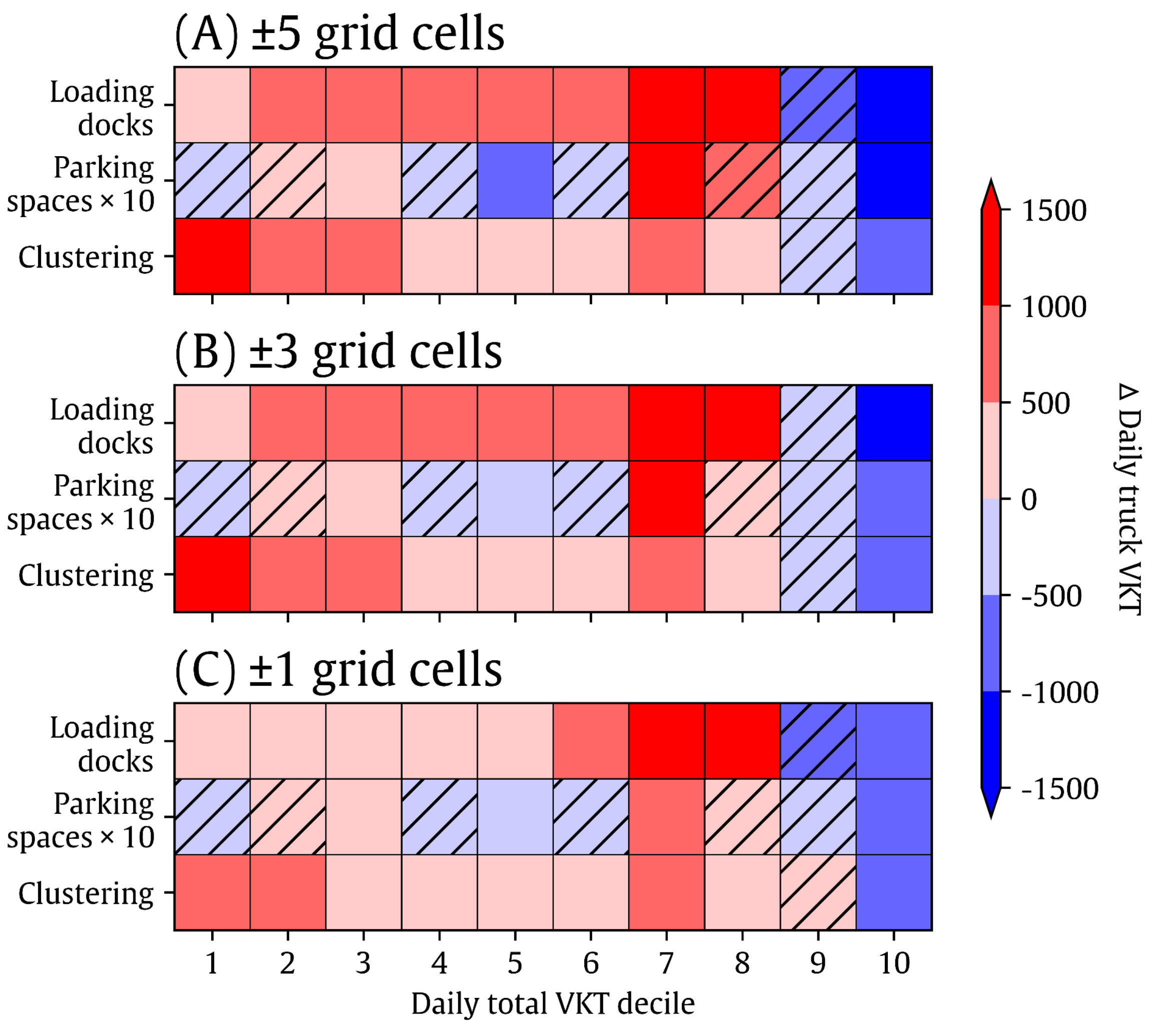

Figure S7. Relationship between warehouse characteristics and truck traffic for different near-warehouse definitions. Slope of the linear regression between daily truck vehicle kilometers traveled (VKT) near warehouses versus the number of loading docks, the number of parking spaces scaled by 10, and clustering for decile bins of daily total VKT near warehouses for daily truck and total VKT within **(A)** ±5 grid cells, **(B)** ±3 grid cells, and **(C)** ±1 grid cells of warehouses. Hatching represents a bin where the relationship between daily truck VKT and a particular warehouse characteristic is not statistically significant.

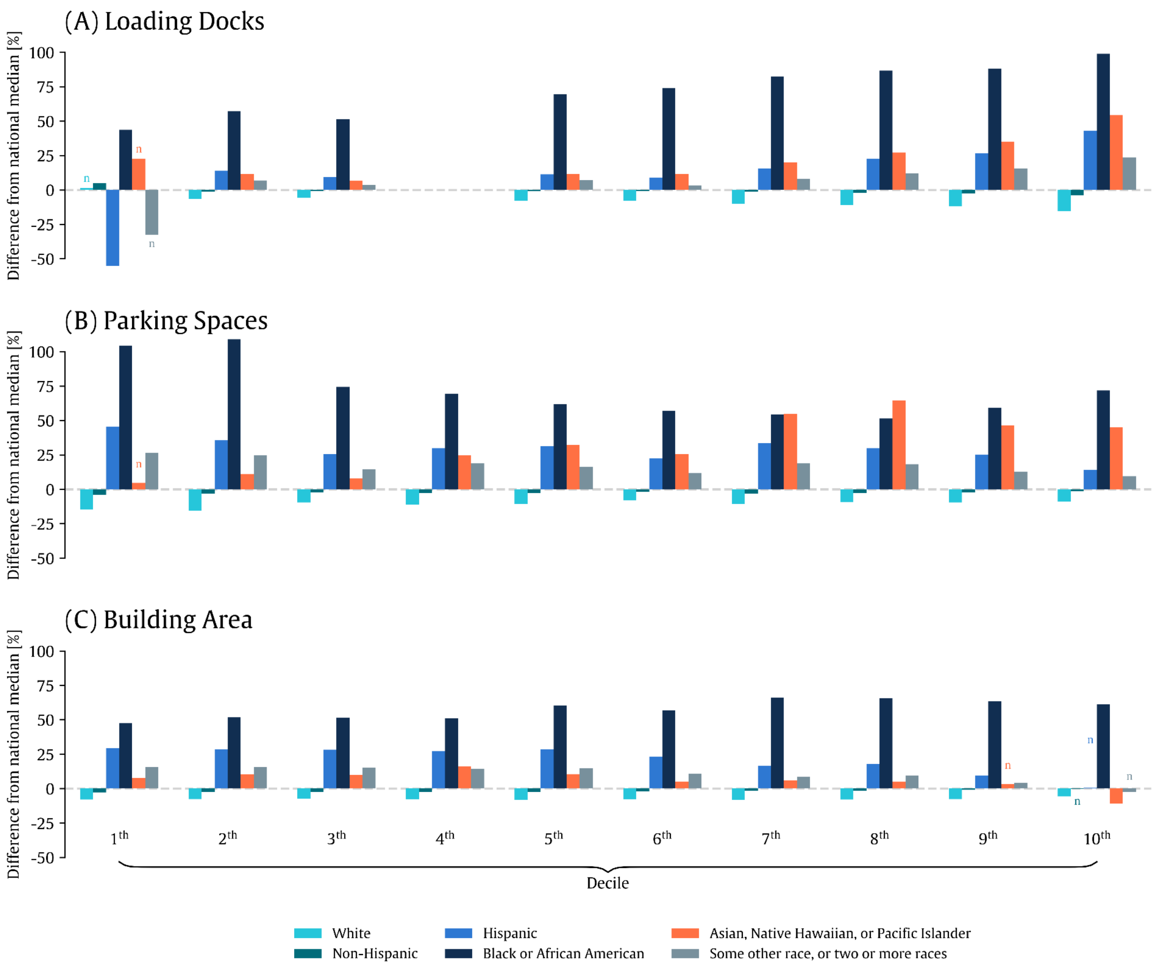

Figure S8. Disparities in the racial-ethnic composition of the near-warehouse population discretized by warehouse characteristics. The relative difference in the median racial and ethnic composition of census tracts containing warehouses and the overall U.S. median demographics for warehouses discretized into deciles based on **(A)** loading docks, **(B)** parking spaces, and **(C)** building area. Bars marked with "n" denote differences between demographics at warehouses and national median demographics that are not statistically significant. Note that the 30th and 40th percentiles (i.e., the fourth decile) of warehouses by their number of loading docks are equal to three docks, yielding no warehouses and associated demographics from which to compute demographic differences.

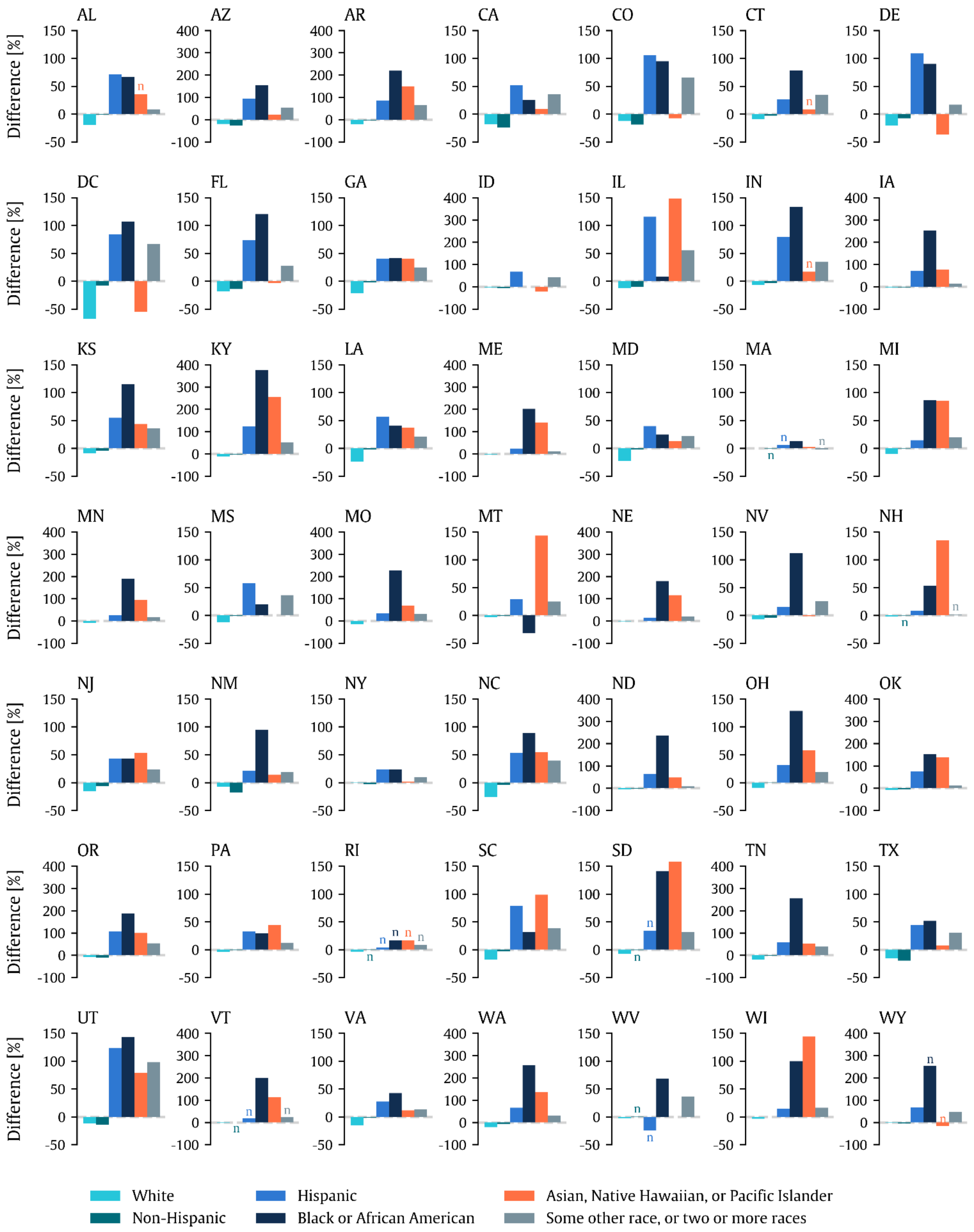

Figure S9. **Disparities in the racial-ethnic composition of the near-warehouse population for individual U.S. states.** The relative difference in the racial and ethnic composition of census tracts containing warehouses in a given U.S. state and the overall median demographics for that state. Bars marked with "n" denote differences between demographics at warehouses and national median demographics that are not statistically significant.

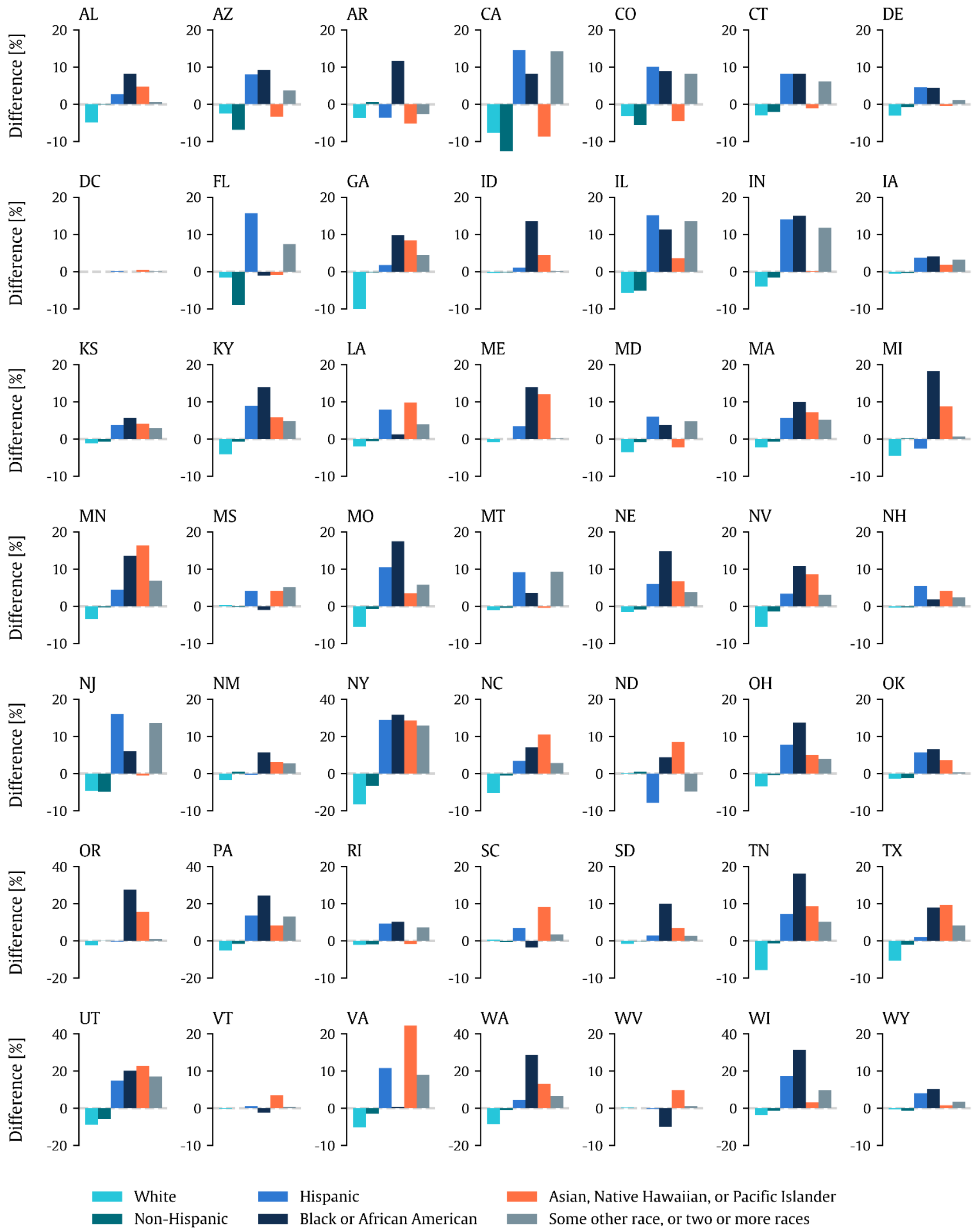

Figure S10. **Disparities in near-warehouse NO_2 for different racial-ethnic populations for individual U.S. states.** The relative difference in population-weighted NO_2 averaged near warehouses in a given U.S. state for different population subgroups and the overall population-weighted NO_2 near warehouses in that state.

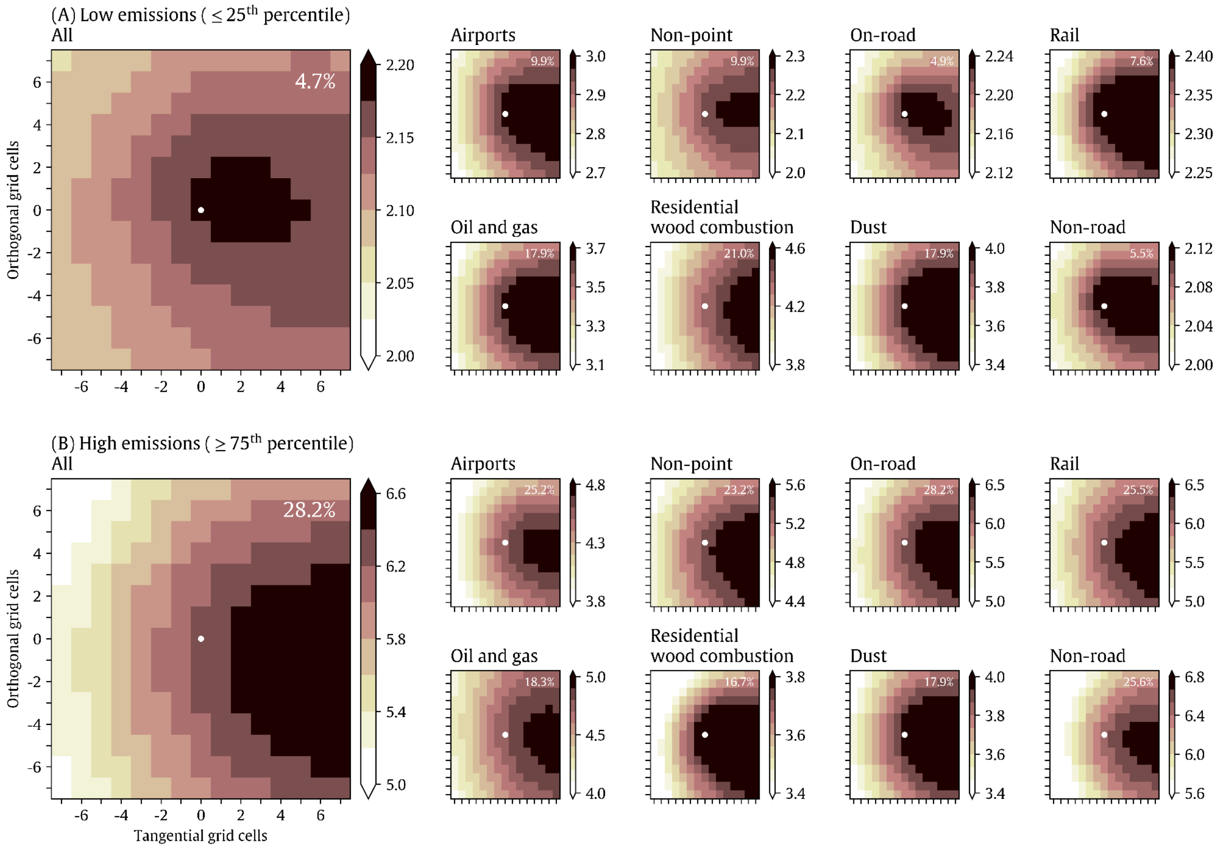

Figure S11. **Near-warehouse NO_2 enhancements for different emission environments.** The annual average 2021 near-warehouse NO_2 signal for warehouses in areas with (A) low and (B) high NO_X emissions given (large panels) all NO_X emissions and (small panels) specific NO_X-emitting sectors. Here, warehouses in low (high) emissions environments are defined as those whose near-warehouse total NO_X or sector-specific NO_X emissions from NEMO are less than the 25th percentile (greater than the 75th percentile) given the distribution of emissions at all warehouses. The white scatterpoint corresponds to the location of the warehouses. The values shown in the composites and defined in the inset colorbars are in units of molecules cm^{-2} and have been scaled by 10^{-15}. Inset text in the upper right corner represents the near-warehouse NO_2 enhancement–that is, the percent NO_2 change between levels at the windward orthogonal edge of the composite and the maximum levels in the composite.

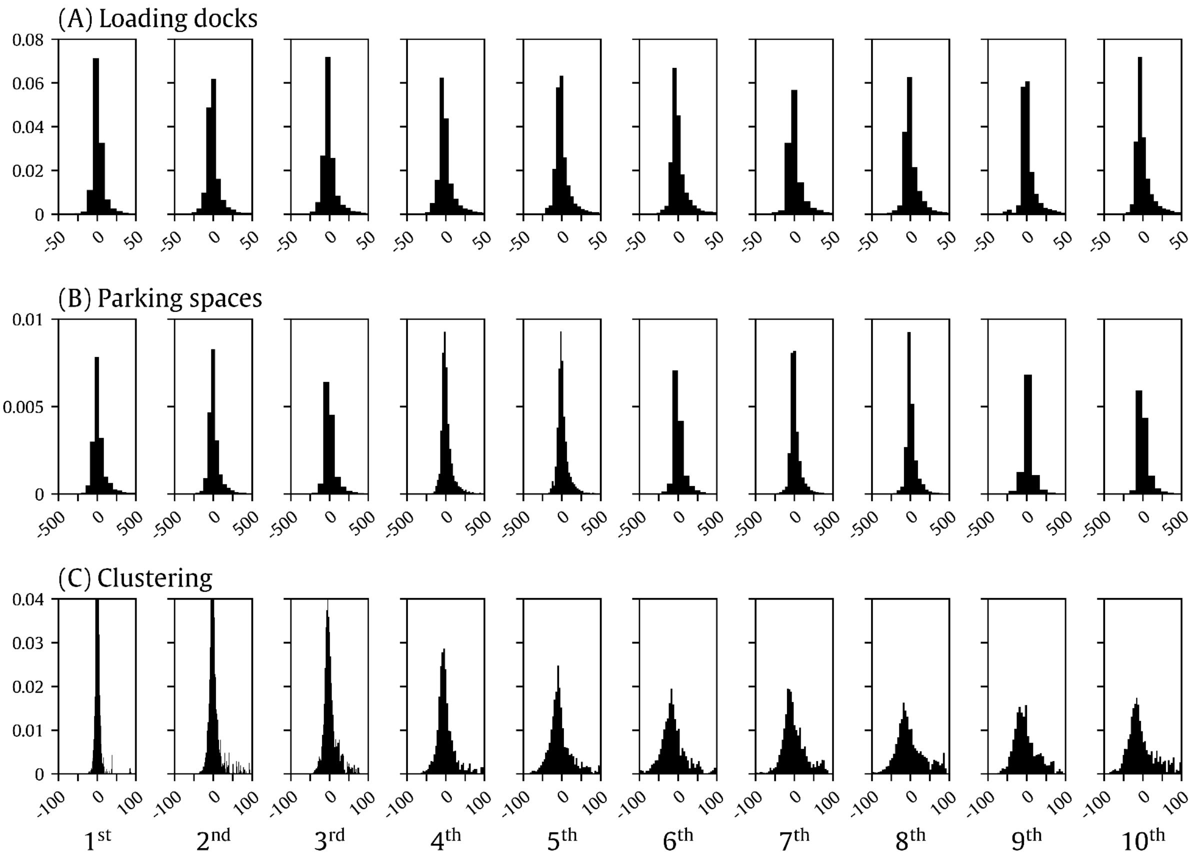

Figure S12. Distribution of residuals of the linear regression between truck VKT and warehouse characteristics. For each decile of total VKT, Figure 3A in the main text shows the slope of the linear regression between warehouse characteristics—specifically **(A)** loading docks, **(B)** parking spaces, and **(C)** clustering—and truck VKT. Here, we show the normalized distribution of the residuals–that is, the difference between the fitted loading dock, parking spaces, or clustering counts and the observed counts–from these linear regressions performed in Figure 3A.

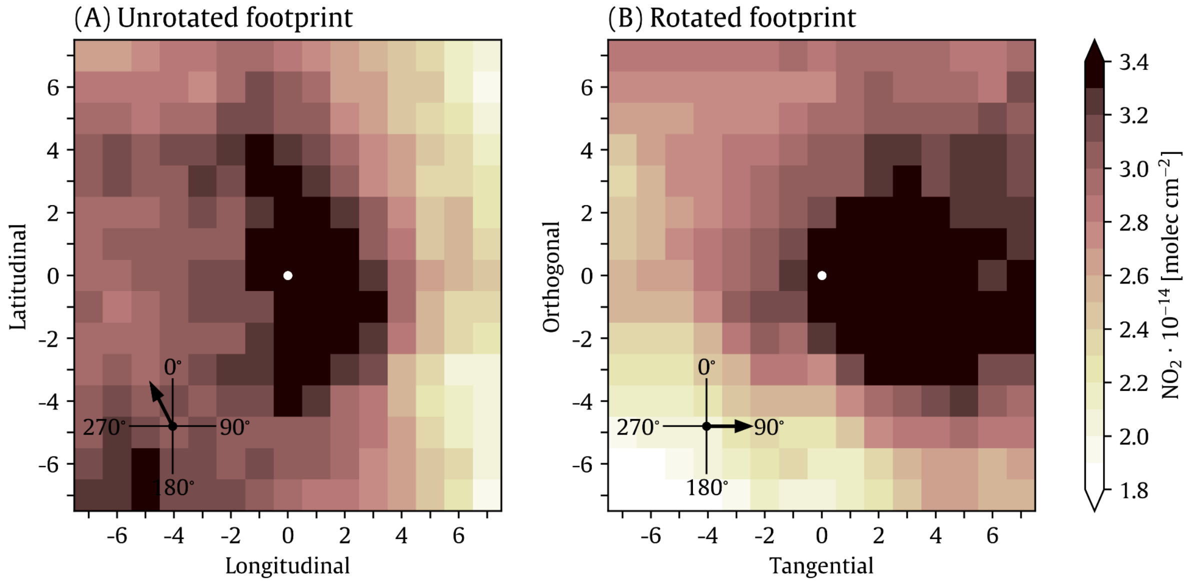

Figure S13. Illustration of near-warehouse NO_2 rotation. (A) Unrotated TROPOMI NO_2 averaged over all days in 2021 near a warehouse, denoted by the white scatterpoint, in census tract 48479001711 in Laredo, Texas. Inset axis denotes the prevailing hourly 100-meter winds averaged over all days (only 16-21 UTC) in 2021. **(B)** TROPOMI NO_2 near the Laredo warehouse artificially rotated such that the artificial prevailing wind direction is to the right of the abscissa in the inset axis.

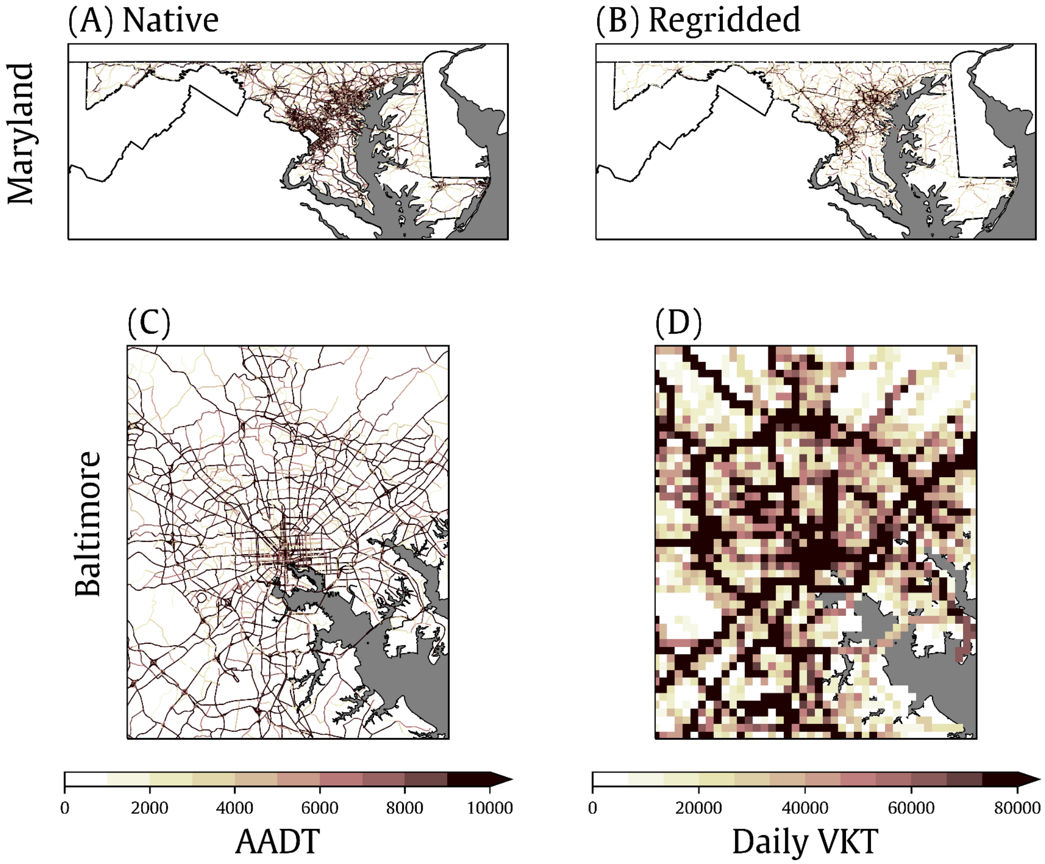

Figure S14. Illustration of transformation of annual average daily traffic (AADT) to daily VKT. Examples are for **(A)-(B)** Maryland and **(C)-(D)** Baltimore City. The spatial resolution of the daily VKT data is 0.01˚ x 0.01˚.
