# Supplementary material for: Air pollution impacts from warehousing in the United States uncovered with satellite data
Source: Nat Commun. 2024 Jul 24;15:6006. doi: 10.1038/s41467-024-50000-0 (PMC11269699; doi:10.1038/s41467-024-50000-0)
Supplement: Supplementary file 3 — Description of Additional Supplementary Files [file 41467_2024_50000_MOESM3_ESM.pdf]

Description of Additional Supplementary Files

File Name: Supplementary Data 1
Description: Total number of warehouses, the number of warehouses per 100,000 population, and the average near-warehouse NO2 enhancement for U.S. states and counties.
